# Supplementary material for: Demographic and Disease-Related Predictors of Socioemotional Development in Children with Neurofibromatosis Type 1 and Plexiform Neurofibromas: An Exploratory Study
Source: Cancers (Basel). 2022 Dec 1;14(23):5956. doi: 10.3390/cancers14235956 (PMC9737030; doi:10.3390/cancers14235956)
Supplement: Supplementary file 1 [file cancers-14-05956-s001.zip › cancers-2015516-supplementary.pdf]

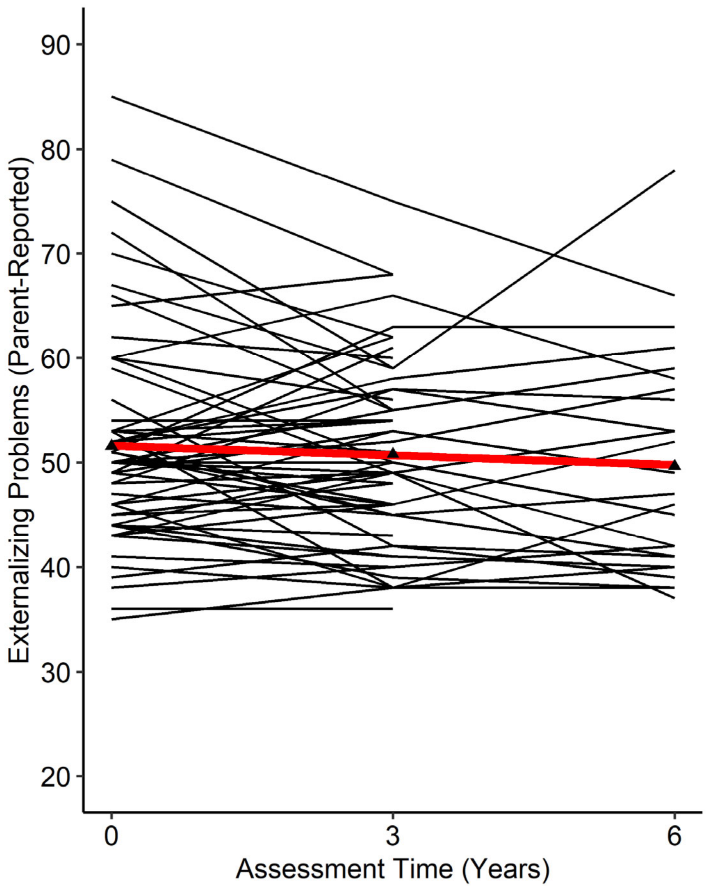

Figure S1. Individual developmental trajectories and estimated average change pattern of externalizing problems (parent-reported). Black lines represent individual trajectories. The red line represents the estimated average developmental trajectory (β = –0.19, *SE* = 0.20, *p* = 0.33). Valid number of participants *n* = 86 and valid number of observations *k* = 167.

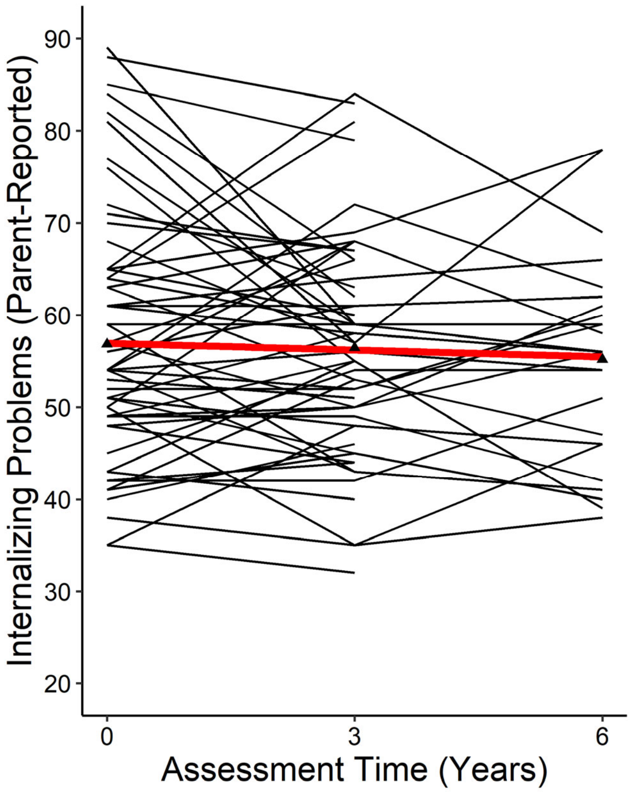

Figure S2. Individual developmental trajectories and estimated average change pattern of internalizing problems (parent-reported). Black lines represent individual trajectories. The red line represents the estimated average developmental trajectory (β = –0.06, *SE* = 0.28, *p* = 0.84). Valid number of participants *n* = 86 and valid number of observations *k* = 167.

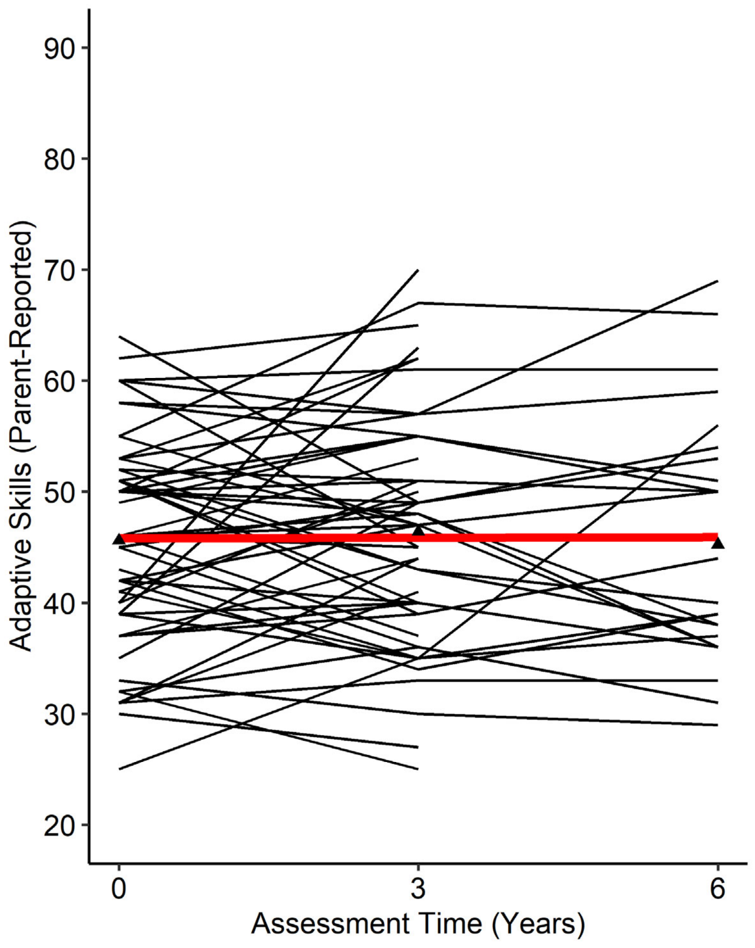

Figure S3. Individual developmental trajectories and estimated average change pattern of adaptive skills (parent-reported). Black lines represent individual trajectories. The red line represents the estimated average developmental trajectory ($\beta = 0.27$, $SE = 0.31$, $p = 0.39$). Valid number of participants $n = 86$ and valid number of observations $k = 167$.

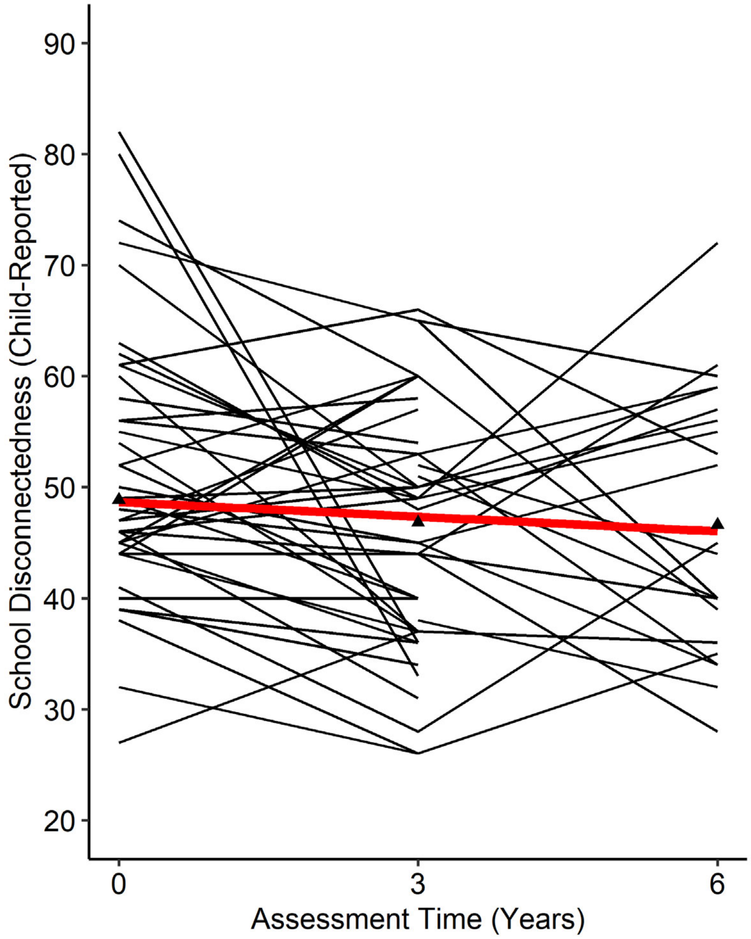

Figure S4. Individual developmental trajectories and estimated average change pattern of school disconnectedness (child-reported). Black lines represent individual trajectories. The red line represents the estimated average developmental trajectory (β = −0.68, *SE* = 0.39, *p* = 0.09). Valid number of participants *n* = 81 and valid number of observations *k* = 142.

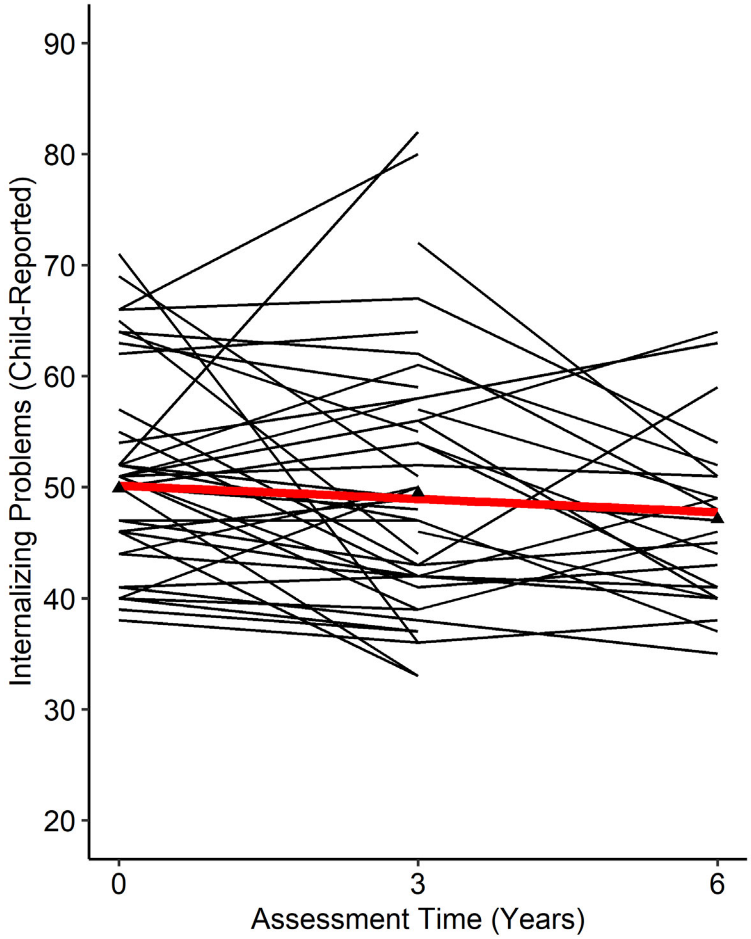

Figure S5. Individual developmental trajectories and estimated average change pattern of internalizing problems (child-reported). Black lines represent individual trajectories. The red line represents the estimated average developmental trajectory (β = −0.53, *SE* = 0.30, p = 0.08). Valid number of participants n = 82 and valid number of observations k = 146.

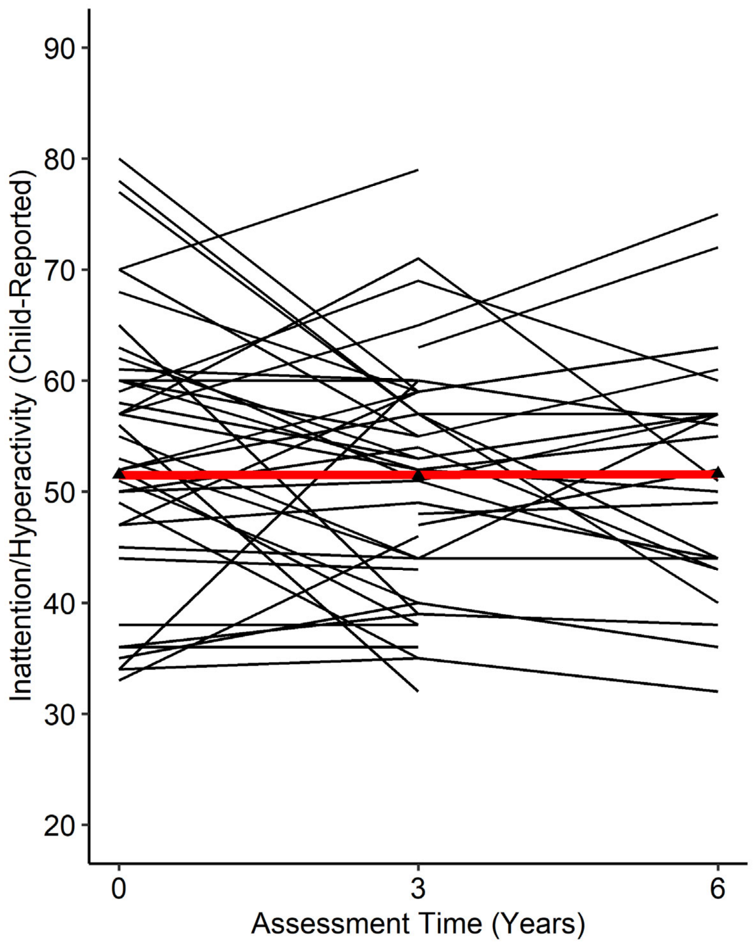

Figure S6. Individual developmental trajectories and estimated average change pattern of inattention/hyperactivity (child-reported). Black lines represent individual trajectories. The red line represents the estimated average developmental trajectory (β = −0.30, *SE* = 0.32, *p* = 0.36). Valid number of participants *n* = 83 and valid number of observations *k* = 147.

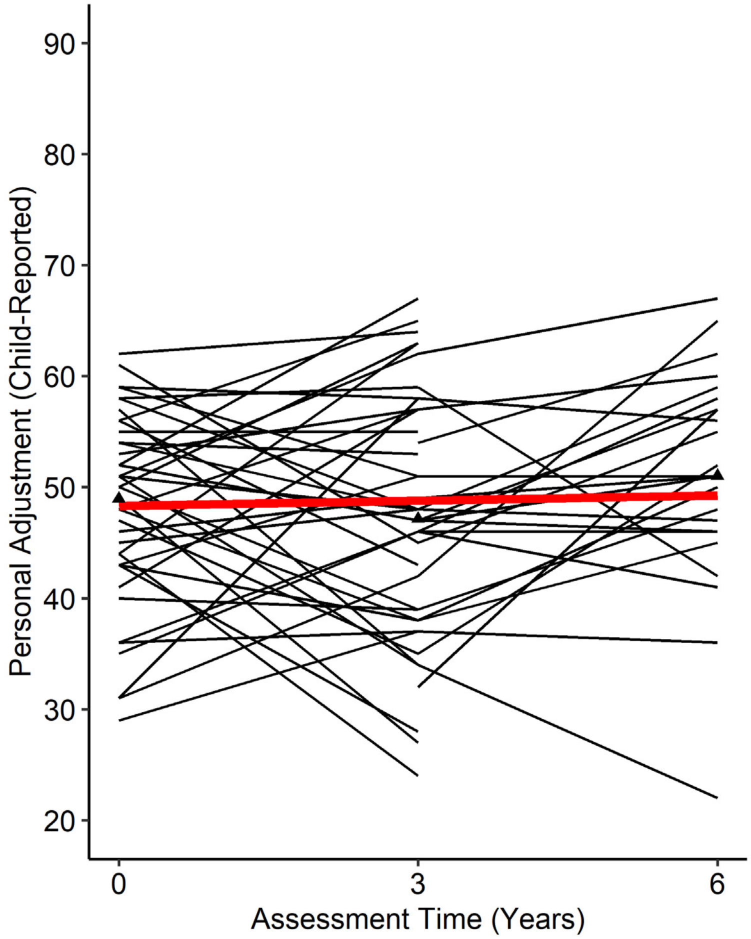

Figure S7. Individual developmental trajectories and estimated average change pattern of personal adjustment (child-reported). Black lines represent individual trajectories. The red line represents the estimated average developmental trajectory (β = 0.30, *SE* = 0.37, *p* = 0.90). Valid number of participants *n* = 82 and valid number of observations *k* = 146.

Table S1. Multilevel Modeling Coefficients of Predictors of Socioemotional Outcomes in the Separate Models.

	Externalizing Problems (PR)		Internalizing Problems (PR)		Adaptive Skills (PR)		School Disconnectedness (CR)		Internalizing Problems (CR)		Inattention/Hyperactivity (CR)		Personal Adjustment (CR)	
Parameters	*β*	*SE*	*β*	*SE*	*β*	*SE*	*β*	*SE*	*β*	*SE*	*β*	*SE*	*β*	*SE*
Intercept	51.39***	1.00	56.93***	1.24	45.66***	1.04	49.68***	1.30	50.20***	1.16	51.80***	1.28	48.16***	1.21
Time	-0.30	0.23	-0.59*	0.30	0.45	0.33	-0.77	0.44	-0.62	0.34	-0.53	0.35	0.28	0.38
Age	-0.44	0.29	0.81*	0.36	0.23	0.30	-1.33**	0.43	0.03	0.37	-0.40	0.41	0.00	0.40
Time × Age	-0.05	0.08	-0.38***	0.10	0.14	0.10	0.27	0.16	-0.07	0.12	-0.09	0.13	-0.02	0.13
Intercept	52.77***	1.55	58.79***	1.91	43.37***	1.56	47.68***	1.97	51.16***	1.74	53.41***	1.93	49.46***	1.79
Time	-0.69*	0.32	-0.48	0.46	0.68	0.50	-0.79	0.65	-0.56	0.49	-0.84	0.51	0.03	0.54
Male	-2.33	2.05	-3.73	2.52	3.99	2.06	1.94	2.62	-1.61	2.29	-3.11	2.56	-2.29	2.38
Time × Male	0.80*	0.40	0.70	0.57	-0.70	0.64	0.13	0.82	0.06	0.63	0.88	0.65	0.47	0.69
Intercept	51.43***	1.01	56.61***	1.23	45.68	1.02	48.78***	1.29	50.28***	1.13	51.70***	1.27	48.12***	1.16
Time	-0.18	0.19	-0.04	0.27	0.23	0.30	-0.66	0.38	-0.55	0.30	-0.31	0.32	0.32	0.34
Pedu	-0.21	0.42	-1.18*	0.51	0.86*	0.42	0.41	0.56	0.24	0.49	0.54	0.55	-0.53	0.50
Time × Pedu	0.26*	0.10	0.40**	0.15	-0.32*	0.15	-0.42*	0.21	-0.23	0.16	-0.15	0.17	0.34	0.18
Intercept	52.81***	2.07	58.33***	2.56	42.43***	2.07	50.19***	2.62	50.92***	2.29	54.53***	2.59	49.38***	2.35
Time	-1.04*	0.44	-1.02	0.63	1.61*	0.64	-2.03*	0.92	-1.16	0.74	-1.48	0.78	-1.26	0.82
White	-1.78	2.38	-2.19	2.94	4.24	2.37	-1.75	3.02	-0.83	2.64	-3.71	2.97	-1.43	2.70
Time × White	1.04*	0.49	1.19	0.70	-1.74*	0.72	1.63	1.01	0.74	0.81	1.40	0.85	1.85*	0.89
Intercept	50.72***	1.21	55.45***	1.49	45.88***	1.25	49.64***	1.58	50.95***	1.37	51.85***	1.53	46.22***	1.40
Time	-0.06	0.23	0.02	0.33	0.40	0.37	-0.91*	0.46	-0.62	0.36	-0.49	0.38	0.65	0.39
SINGLP	2.38	2.21	3.99	2.71	-0.81	2.27	-2.72	2.80	-2.25	2.43	-0.65	2.74	6.18*	2.49
Time × SINGLP	-0.51	0.45	-0.26	0.64	-0.47	0.72	0.79	0.88	0.26	0.68	0.70	0.72	-1.13	0.74

Note. *β* = regression coefficients in multilevel growth models; SE = standard error; Pedu = parental education; SINGLP = single parent; NF1= Neurofibromatosis type 1; PNF1 = parental NF1 status; Visi = visibility of tumors; Seve = severity of NF1 symptoms; Comp = NF1-related disease complications; PR = parent-reported; CR = child-reported. * $p \leq .05$; ** $p < .01$; *** $p < .001$. Valid number of participants (n) and valid number of observations (k) for the seven models were: externalizing problems (PR), n = 86, k = 167; internalizing problems (PR), n = 86, k = 167; adaptive skills (PR), n = 86, k = 167; school disconnectedness (CR), n = 81, k = 142; internalizing problems (CR), n = 82, k = 146; inattention/hyperactivity (CR), n = 83, k = 147; personal adjustment (CR), n = 82, k = 146.

Table S1 (*Continued*)

Parameters	Externalizing Problems (PR) β	SE	Internalizing Problems (PR) β	SE	Adaptive Skills (PR) β	SE	School Disconnectedness (CR) β	SE	Internalizing Problems (CR) β	SE	Inattention/Hyperactivity (CR) β	SE	Personal Adjustment (CR) β	SE
Intercept	49.81***	1.47	55.51***	1.84	46.78***	1.52	48.07***	1.90	48.73***	1.62	51.23***	1.79	48.09***	1.71
Time	-0.07	0.26	0.22	0.36	0.29	0.41	-0.94	0.52	-0.43	0.40	-0.41	0.42	0.72	0.45
PNF1	3.58	2.16	3.74	2.71	-2.29	2.23	1.92	2.79	4.43	2.42	1.51	2.65	-1.42	2.51
Time × PNF1	-0.29	0.42	-0.79	0.58	0.08	0.64	0.78	0.83	-0.36	0.65	0.22	0.67	-0.82	0.71
Intercept	51.35***	1.55	54.24***	1.89	48.65***	1.53	45.68***	1.99	48.18***	1.73	50.95***	1.97	47.73***	1.84
Time	0.09	0.29	0.19	0.41	-0.71	0.45	0.17	0.56	-0.39	0.46	-0.06	0.49	0.40	0.51
Visi	0.17	2.05	4.24	2.51	-5.30*	2.02	5.31*	2.60	3.52	2.27	1.20	2.58	0.75	2.40
Time × Visi	-0.52	0.40	-0.44	0.56	1.74**	0.60	-1.54*	0.77	-0.24	0.61	-0.44	0.65	-0.16	0.68
Intercept	51.15***	1.78	49.75***	2.06	49.32***	1.76	49.03***	2.27	47.72***	1.95	50.49***	2.22	48.31***	2.06
Time	0.32	0.34	0.65	0.47	-0.66	0.55	-0.32	0.65	-0.44	0.52	0.10	0.55	0.92	0.57
Seve	0.46	2.17	10.25***	2.51	-5.47*	2.15	-0.35	2.77	3.72	2.38	1.74	2.71	-0.15	2.51
Time × Seve	-0.77	0.41	-1.08	0.58	1.34*	0.66	-0.56	0.81	-0.14	0.64	-0.61	0.68	-0.92	0.70
Intercept	51.42***	1.00	56.71***	1.22	45.64	1.04	48.75***	1.30	50.23***	1.13	51.63***	1.26	48.14***	1.18
Time	-0.15	0.19	-0.04	0.28	0.24	0.31	-0.67	0.39	-0.52	0.31	-0.30	0.32	0.34	0.33
Comp	-0.07	0.59	1.96**	0.71	-0.23	0.61	-0.71	0.75	0.70	0.65	0.78	0.73	0.51	0.69
Time × Comp	-0.37**	0.13	-0.34	0.18	0.25	0.20	-0.01	0.25	-0.14	0.20	-0.04	0.21	-0.28	0.22

Note. β = regression coefficients in multilevel growth models; *SE* = standard error; Pedu = parental education; SINGLP = single parent; NF1= Neurofibromatosis type 1; PNF1 = parental NF1 status; Visi = visibility of tumors; Seve = severity of NF1 symptoms; Comp = NF1-related disease complications; PR = parent-reported; CR = child-reported. $^{*}p \leq .05$; $^{**}p < .01$; $^{***}p < .001$. Valid number of participants (*n*) and valid number of observations (*k*) for the seven models were: externalizing problems (PR), n = 86, k = 167; internalizing problems (PR), n = 86, k = 167; adaptive skills (PR), n = 86, k = 167; school disconnectedness (CR), n = 81, k = 142; internalizing problems (CR), n = 82, k = 146; inattention/hyperactivity (CR), n = 83, k = 147; personal adjustment (CR), n = 82, k = 146.

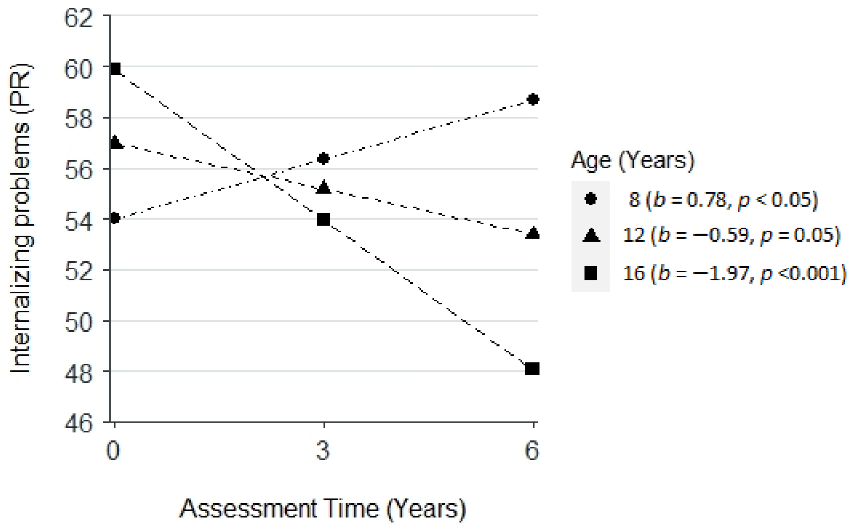

Figure S8. Plot for the interaction effect among assessment time and age at baseline on internalizing problems (parent-reported, PR). Three levels of baseline age were plotted: 8 years (1 SD below the mean), 12 years (the mean), and 16 years (1 SD above the mean).

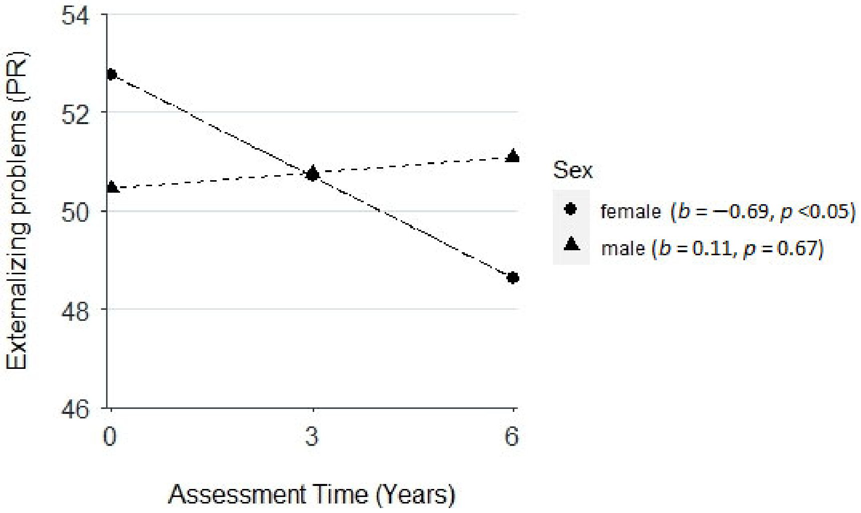

Figure S9. Plot for the interaction effect among assessment time and sex on externalizing problems (parent-reported, PR).

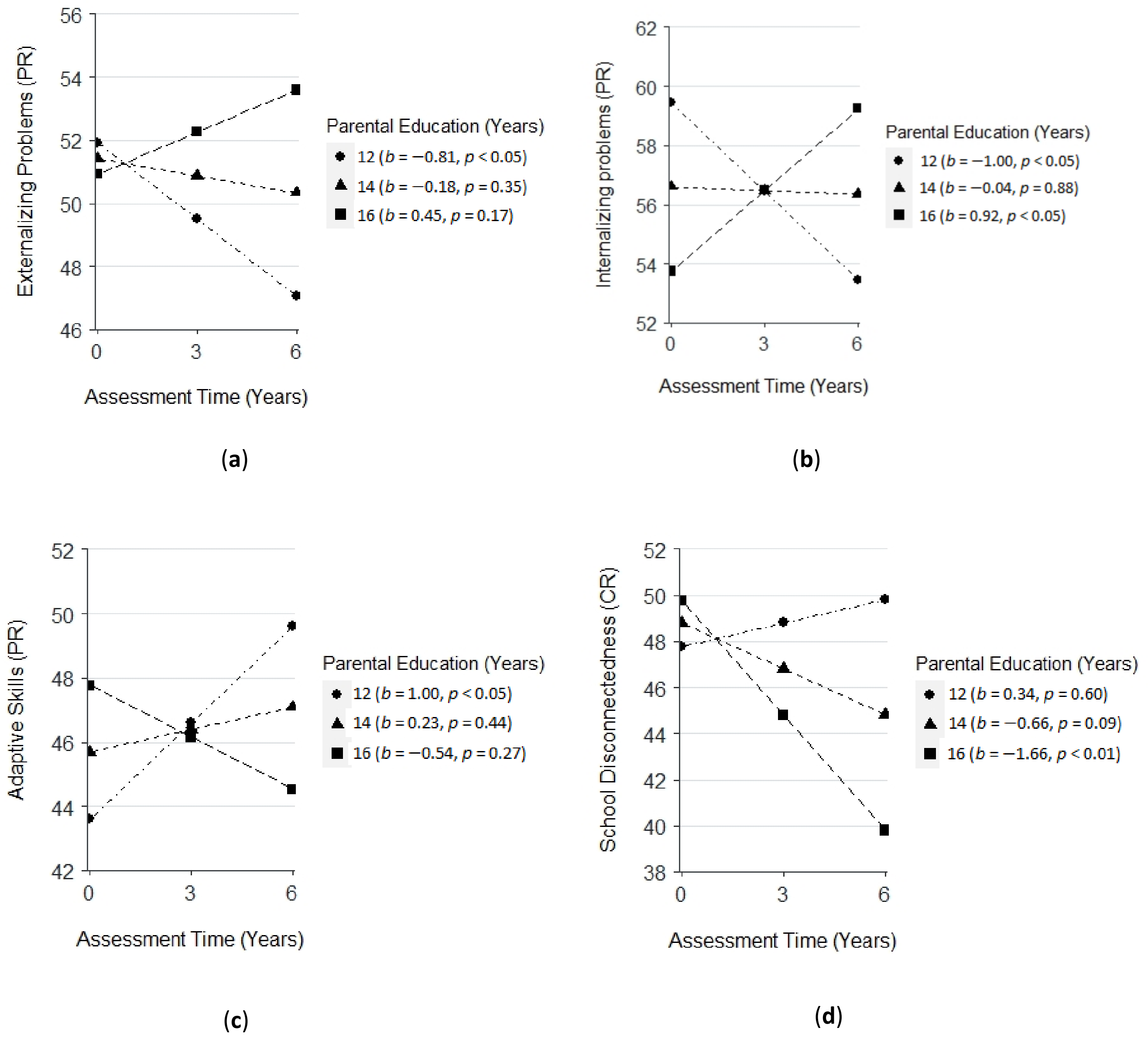

Figure S10. Plot for the interaction effect among assessment time and parental education on (**a**) externalizing problems (parent-reported, PR), (**b**) internalizing problems (parent-reported, PR), (**c**) adaptive skills (parent-reported, PR), (**d**) school disconnectedness (child-reported, CR). Three levels of parental education were plotted: 12 years (1 SD below the mean), 14 years (the mean), and 16 years (1 SD above the mean).

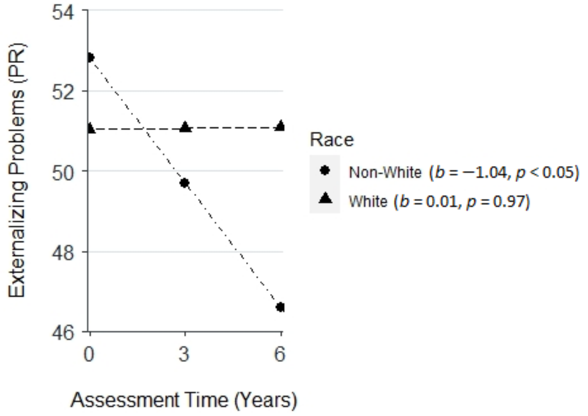

(a)

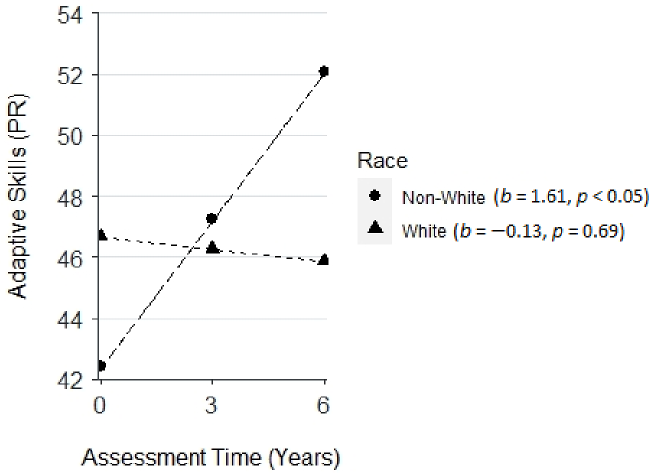

(b)

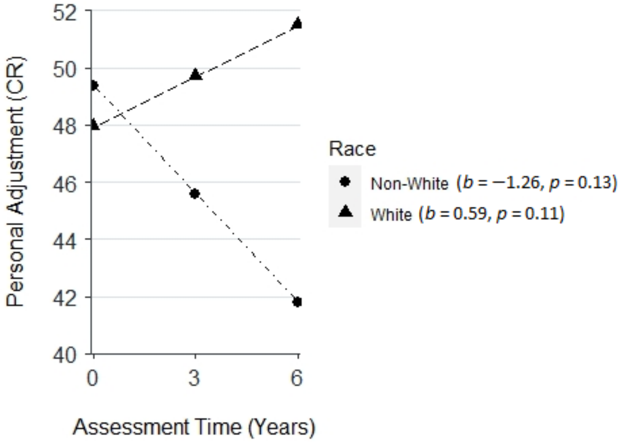

(c)

Figure S11. Plot for the interaction effect among assessment time and race on (**a**) externalizing problems (parent-reported, PR), (**b**) adaptive skills (parent-reported, PR), (**c**) personal adjustment (child-reported, CR).

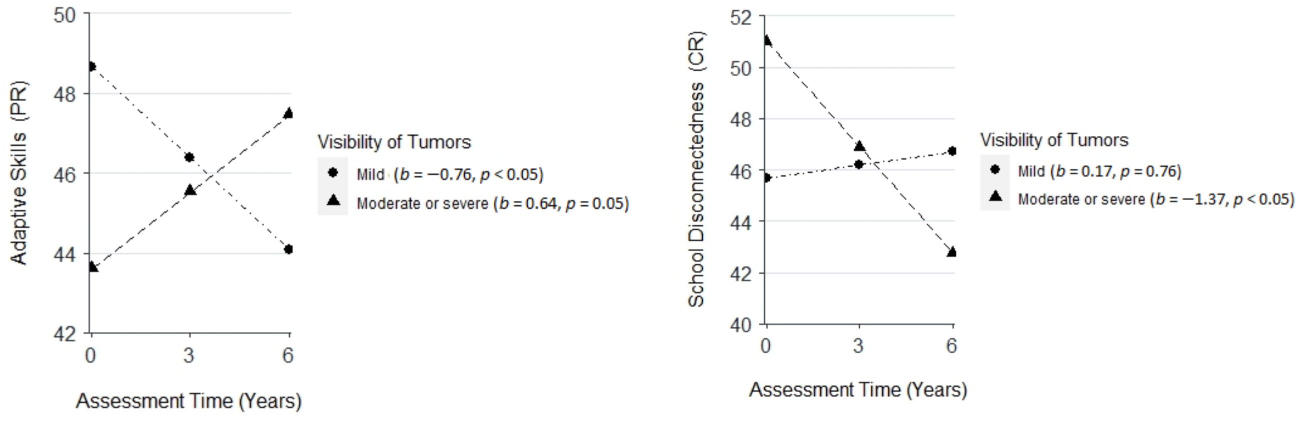

(a) (b)

Figure S12. Plot for the interaction effect among assessment time and visibility of tumors on (**a**) adaptive skills (parent-reported, PR), (**b**) school disconnectedness (child-reported, CR).

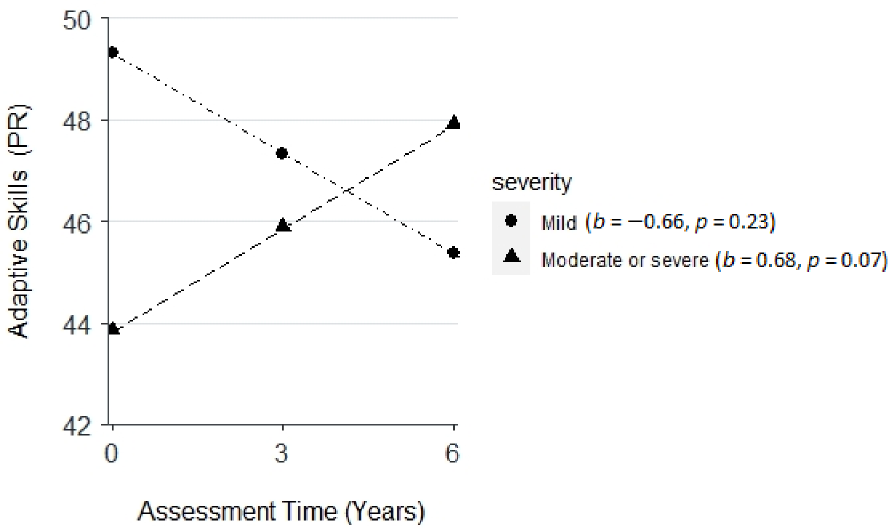

Figure S13. Plot for the interaction effect among assessment time and NF1 disease severity on adaptive skills (parent-reported, PR).

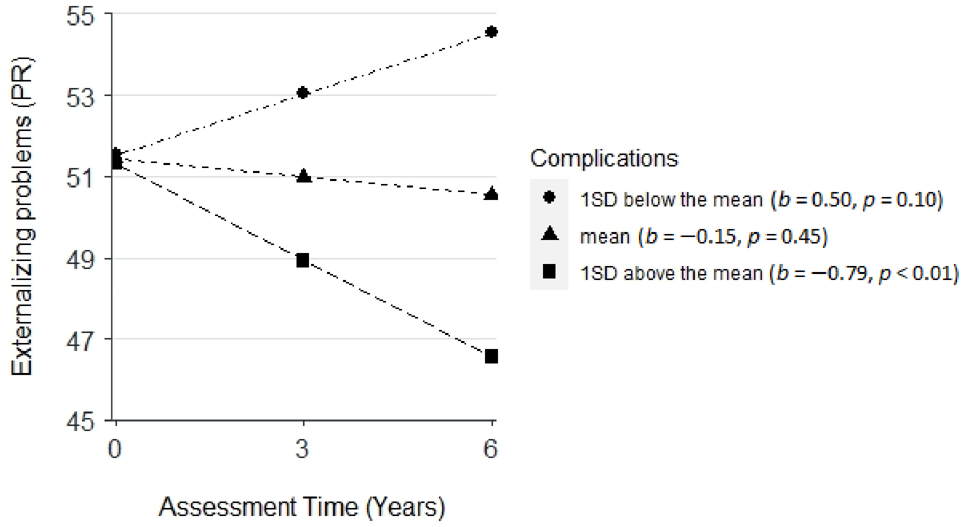

Figure S14. Plot for the interaction effect among assessment time and NF1-related disease complications on externalizing problems (parent-reported, PR).
